# Typhoid Fever and Sanitation

**Published:** 1903-04-11

**Authors:** 


### TYPHOID FEVER AND SANITATION.

There is always one great obstacle in the way of the would-be sanitary reformer whose aims are in any degree comprehensive, namely, the expense which his schemes will, if carried out, entail upon the community concerned. There must of necessity be a doubt in the mind of the ordinary citizen whether the outlay will be ultimately balanced by the result. It is for this reason highly important to be able to produce specific evidence to prove in the case of sanitary improvements carried out on a large scale that wisdom is justified of her children. At the meeting of the Intercolonial Medical Congress in Melbourne in 1889 the subject of typhoid fever was under consideration, and the undue prevalence of the illness in Melbourne itself was discussed, and in the end three resolutions were proposed and carried unanimously. The first affirmed: "That the prevalence of typhoid fever is mainly owing to insanitary conditions, and above all to contaminated water supply, defective drainage, and improper disposal of night soil." The second, "That while there is reason to believe that the sources of the water supply of Melbourne are carefully guarded, it is certain that, as regards drainage and night soil disposal, the arrangements are very unsatisfactory, and to these defects must be ascribed in great measure the excessive prevalence of typhoid fever year after year." By the third resolution it was declared, "That in the opinion of this Congress, it is the imperative duty of the Government to take immediate steps for bringing about an improvement in the sanitary condition of Melbourne, and specifically for the construction of a proper system of underground drainage, which shall include the removal of nightsoil by water carriage." Dr. James Jamieson in a paper published in the *Australasian Medical Gazette* shows what has been achieved in Melbourne towards diminishing the prevalence of typhoid fever in that city since those resolutions were passed. In 1890 a Metropolitan Board of Works was constituted with control of water supply and drainage. In 1897 house connections began to be made with a main sewerage system and by August 1902, 48,000 buildings out of about 100,000 had been connected with the sewers, and the pan system abolished so far as these buildings were concerned. Dr. Jamieson gives a table setting forth the mortality rate from typhoid fever in Melbourne per 10,000 of the living population. From this table it is seen that already an appreciable reduction has taken place in the mortality from enteric fever in Melbourne. Moreover he shows that in the years 1900 and 1901 the death rate from typhoid has been lower in Melbourne itself than in the rest of Victoria, though such a thing had not occurred before in all the years under review (1890 to 1901). As Dr. Jamieson points out a lowering of the death rate by even one per 10,000 of population represents in a place like Melbourne, with a population of about half a million, 50 lives saved every year and probably about 500 fewer cases of enteric. The value of 50 lives of persons in the prime of life, as the majority of typhoid patients are, and the cost of 500 cases of tedious illness each year are not matters which can be dismissed as trifles. By themselves, in fact, they make in their saving a considerable offset against the expense of sewerage; and when to these savings there is added the comfort of living in a sewered house it may well be a question whether the offset is not a full one, and this without taking into account any other affections than typhoid fever, the prevalence of which will also fall with improved methods of sanitation.

